# Rapid resource depletion on coral reefs disrupts competitor recognition processes among butterflyfish species

**DOI:** 10.1098/rspb.2022.2158

**Published:** 2023-01-11

**Authors:** S. A. Keith, J-P.A. Hobbs, L. Boström-Einarsson, I. R. Hartley, N. J. Sanders

**Affiliations:** ^1^ Lancaster Environment Centre, Lancaster University, Lancaster LA1 4YQ, UK; ^2^ School of Biological Sciences, The University of Queensland, Brisbane, QLD 4069, Australia; ^3^ Department of Ecology and Evolutionary Biology, University of Michigan, Ann Arbor, MI 48109, USA

**Keywords:** contest, signalling, *Chaetodon*, coral bleaching, environmental change, behavioural macroecology

## Abstract

Avoiding costly fights can help conserve energy needed to survive rapid environmental change. Competitor recognition processes help resolve contests without escalating to attack, yet we have limited understanding of how they are affected by resource depletion and potential effects on species coexistence. Using a mass coral mortality event as a natural experiment and 3770 field observations of butterflyfish encounters, we test how rapid resource depletion could disrupt recognition processes in butterflyfishes. Following resource loss, heterospecifics approached each other more closely before initiating aggression, fewer contests were resolved by signalling, and the energy invested in attacks was greater. By contrast, behaviour towards conspecifics did not change. As predicted by theory, conspecifics approached one another more closely and were more consistent in attack intensity yet, contrary to expectations, resolution of contests via signalling was more common among heterospecifics. Phylogenetic relatedness or body size did not predict these outcomes. Our results suggest that competitor recognition processes for heterospecifics became less accurate after mass coral mortality, which we hypothesize is due to altered resource overlaps following dietary shifts. Our work implies that competitor recognition is common among heterospecifics, and disruption of this system could lead to suboptimal decision-making, exacerbating sublethal impacts of food scarcity.

## 1. Background

The costs of interference competition can be high, leading to the loss of resources, decreased vigilance, energy depletion or death [1–3]. Mechanisms to avoid, or at least minimize, those costs not only affect individual survival but can scale up to mediate the size, coexistence and spatial distributions of populations [2,4]. By recognizing a competitor, individuals can make decisions about whether to escalate, or retreat from, a potential contest, thereby conserving energy and avoiding injuries [5,6]. Furthermore, as human-induced changes to environmental conditions cause more frequent and severe reductions in resources, the costs and benefits of such interactions are likely to change [7].

Natural selection should favour individuals that are able to identify and discriminate the competitive threat posed by individuals of different classes (e.g. species, age and sex) and respond to that individual in a way that optimizes the net payoff from any interaction [5], a process known as competitor recognition [3,8]. This process requires the expression of a visual, acoustic, chemical or even electrical phenotypic cue that recipients can recognize by comparison with a neural template [1,3,8,9]. The process concludes with a behavioural response—if recognized as a competitor, the receiver can avoid, signal or attack [3]. Competition over limited resources should be most intense between individuals with the highest resource overlap, which are usually conspecifics [4,7,10,11]. Moreover, competitor recognition processes are expected to decrease in precision as interacting species overlap less in resource use and increase in phylogenetic distance [10,12]. However, it is unclear to what extent these ideas are supported empirically, and how it applies to diverse ecological communities with high resource overlap among species, such as those found on coral reefs [4,13,14].

Theoretical expectations for how competitor recognition systems operate within real-world, complex communities are scarce in the literature [3]. The Uncertainty Hypothesis proposed by Peiman & Robinson [10] posits that individuals should be better able to assess defeat probabilities and decide when it is optimal to attack against conspecifics, compared to heterospecifics, due to greater familiarity with conspecific signals and displays, and more certainty about costs due to shared fighting strategies and weaponry. Peiman & Robinson [10] suggest four predictions with which to test this hypothesis empirically: (i) conspecifics are willing to approach one another more closely than heterospecifics; (ii) conspecifics are more likely to resolve a contest from signalling without the need to escalate to attack; (iii) conspecific attack intensity will be less variable than attacks between heterospecifics and (iv) with increasing phylogenetic distance between heterospecifics we would observe a reduced willingness to approach, a reduction in signalling relative to attacks to resolve contests and increasingly variable attack intensity. Body size differences between species can also be used to resolve conflict without the need to escalate to physical fights by avoidance or signalling [10]. If this were the case, we would expect heterospecifics with the largest body size differences to approach each other less closely and for agonistic interactions to be less likely to escalate to fights. Therefore, we also test whether body size differences between heterospecifics are predictive of the proportion of encounters that lead to attacks.

Rapid environmental change could alter the competitive landscape and affect the efficacy of competitor recognition processes, from cue to response, with important ramifications for coexistence [6,10]. However, there are no formal hypotheses for how this process is expected to change under rapid resource loss. Guild-wide dietary shifts can occur after sudden disturbance events (e.g. [15]) with the potential to alter dietary overlap between species. It is unclear to what extent recognition processes can keep pace with this shift. Furthermore, the cognitive load required to discriminate under novel conditions might be higher, which could reduce the sensory capacity available to monitor other threats [16]. Such suboptimal responses could lead to detrimental loss of energy through unnecessary aggression with knock-on effects for population dynamics, species coexistence and even speciation processes [2,17].

Efforts to understand how competitor recognition processes could scale up to impact populations, communities and ecosystems, and how this could be disrupted by environmental change, have been limited for three reasons [18]. First, most studies are restricted to two or three species systems ([19,20], but see [21]), while most ecological communities consist of dozens or more species. Second, laboratory studies cannot simulate realistic environmental change, yet competitor recognition systems are difficult to identify directly in the field. Third, the generalizability of findings can be restricted because field limitations constrain data collection to one location. One way forward is to use patterns in the frequency and intensity of agonistic behaviours across a guild to infer competitor recognition processes [21,22], with a macroecological approach that collects data from multiple locations over a large geographical extent to ensure conclusions are generalizable (e.g. [7]).

Here, we test the predictions of Peiman & Robinson [10] using 3770 observations of coral-feeding butterflyfish encounters between 32 species across 17 reefs, before and after a mass coral mortality event. Butterflyfishes reduced aggression and shifted diets in response to rapid reductions in resource availability following the 2016 global mass bleaching event where coral—the major food resource of butterflyfishes—decreased by 18–65% of initial cover [7,15]. This response was replicated at multiple locations, providing a natural experiment to explore the effect of environmental change on competitor recognition processes.

## 2. Methods

### (a) Study system

#### (i) Taxon

Coral reef butterflyfishes (genus *Chaetodon*) offer a model field system to examine how changes in resource availability can affect competitor recognition processes for several reasons. First, butterflyfishes show clear signalling (e.g. tail-up while head angled down, erection of the dorsal fin) and attacks during both conspecific and heterospecific encounters [23,24], and changes in resource availability can lead to changes in aggression both within and among species [7,25]. Second, butterflyfishes use predominantly visual cues for species recognition [26–28]. Third, individuals of many butterflyfish species form stable mated pairs [24], meaning that conspecific aggression is overwhelmingly driven by access to food rather than reproduction. Fourth, the phylogeny of butterflyfishes is well resolved [29], which allows us to test the hypothesis that behavioural responses involved in the competitor recognition process can be predicted by phylogenetic relatedness of species [4,8].

#### (ii) Field sites

We observed encounters among butterflyfishes at five regions across the central Indo-Pacific: Iriomote (Japan; 123.7°E, 24.4°N), Christmas Island (Indian Ocean; 105.6°E, 10.4°S), Luzon (the Philippines; 120.8°E, 13.7°N), Aceh (Indonesia; 95.1–95.3°E, 5.4–5.9°S) and Bali (Indonesia; 115.6°E, 8.4°S). We visited reefs up to 12 months either side of a global mass coral bleaching event [7]. We recorded data on 17 reefs in total, comprising 3–4 sampled reefs per region. Reefs were separated by greater than 1 km non-reef patches (corallivorous butterflyfish territories are generally less than 0.2 km^2^) [30] to sample different populations. For further information on field sites and data collection, see Keith *et al.* [7].

### (b) Data collection

We used a well-established protocol to observe butterflyfishes on snorkel or SCUBA depending on depth and visibility [31,32]. Focal individuals were followed at a distance of 2–4 m for 5 min following an acclimation period (approx. 1 min) to check that the individual was responding naturally (that is, feeding). Butterflyfish are often found in a mated pair, so to ensure independence of sampled individuals, only one individual of each pair was recorded. To reduce the risk of selecting the same fish as a focal individual twice, we used a U-shaped search pattern and attempted to observe one individual from every pair present on the reef. When a congeneric individual from outside the mated pair came within 1 m of the focal individual, we assumed they were aware of each other's presence with the potential to interact and therefore recorded an encounter.

Proximity was recorded as the smallest distance observed between two individuals during an encounter. Encounter outcomes were recorded as passive, where neither individual showed a discernible change in behaviour, or aggressive. Aggressive encounters were further subdivided into those that involved signalling only and those that escalated to a chase [32]. Both forms of aggression are linked strongly to competition over food resources, and as the majority of our observed individuals were in a pair, the possibility that they were engaging in courtship displays with individuals outside of their pair was minimal [32]. Each new observer underwent training by an experienced observer (either J-P.H. or S.A.K.) until recorded data were identical to ensure standardization. Behaviour was unlikely to have been affected by diver presence [33].

### (c) Statistical analysis

Data used for analysis were restricted to species pairs with at least five encounters across five different focal individuals. For some analyses, data were restricted further to include only species that were present with these minimum sample sizes in both conspecific and heterospecific encounters. This reduced dataset ensured that any differences were not driven solely by a larger and more variable pool of species in the heterospecific encounters. Note that due to this requirement to use a reduced dataset, statistical models with more parameters (e.g. generalized linear mixed effects models) were not appropriate. The three Philippines reefs did not experience significant coral mortality as a result of bleaching and are therefore not included in the data for after the coral mortality event. All analyses were done in R v.3.6.1. [34].

*Prediction 1: Individuals approach heterospecifics less closely than conspecifics.* Proximity distances were estimated to the nearest 25 cm in the field (0–24 cm, 25–49 cm, 50–74 cm and 75–100 cm) and converted to dummy variables (1–4) for analysis. To account for non-independence of repeated samples, we calculated the mean of the distance categories from the dummy variables for each individual across its conspecific and heterospecific encounters separately. Use of the mean is appropriate for ordinal data in this case because the numeric difference between each category is equal. We then used a permutation-based two-tailed Mann–Whitney U test from the coin package [35] with a Monte Carlo-derived approximate distribution to determine whether the mean proximity during conspecific encounters was significantly different from the mean proximity during heterospecific encounters. Data were logged for plotting purposes only.

*Prediction 2: Signalling is more common between conspecifics than heterospecifics*. We calculated the proportion of aggressive encounters that involved visual signalling for conspecific and heterospecific pairs both before and after bleaching. To deal with non-independence of samples due to repeated measures within individuals (i.e. multiple encounters per individual), we bootstrapped the data 1000 times, each time sampling one encounter only per individual. For each bootstrapped dataset, we tested whether the frequency of signalling (rather than escalation to attack) for conspecific and heterospecific encounters, and before and after coral mortality within those groups, were significantly different from expected using chi-squared permutation tests from the coin package [35], which is robust to small or skewed sample sizes. We calculated the mean chi-squared statistics and *p*-values, and their 95% confidence intervals, across bootstrapped datasets.

*Prediction 3: Attack intensity is more variable between heterospecifics than conspecifics*. We used the coefficient of variation (CV) to quantify variation in chase distances across heterospecific and conspecific encounters relative to the mean. We use this approach because the mean chase distance is higher for conspecifics, as we would expect from the literature and theory, so a measure *relative to the mean* is essential. To minimize the influence of rare long chases, we grouped all that were ≥ 10 m. We tested whether there was a significant difference in the CV between conspecific and heterospecific chase distances using the modified signed-likelihood ratio test for equality of CVs (MLSR), which is robust to differences in sample size [36], from the R package cvequality [37].

*Prediction 4: Proximity, signalling and variation in attack intensity can be predicted by phylogenetic relatedness and difference in body size.* To determine whether approach proximity, signalling proportion and variation in chase distance could be predicted by phylogenetic relatedness, we used branch length between each species pair in the phylogeny [29], calculated with the ape package [38]. We also tested whether body size could predict these behaviours because it can be a cue for individuals to identify competitors. Body size differences were calculated from the species level trait maximum body length downloaded from Fishbase [39], which is appropriate because Chaetodontids achieve 68–92% of their full adult body size in the first year and do not differ perceptibly as adults [40]. Conspecifics were excluded to ensure the result could be interpreted as a nuanced representation and was not overwhelmed by zeros (i.e. for phylogenetic distance). We generated separate regression models for mean proximity, signalling proportion and variation in chase distance as dependent variables (species pairs: *n* = 107 proximity; *n* = 24 signalling; *n* = 16 for chase variation) and checked QQ plots to ensure assumptions were met for proximity and attack intensity. Signalling was modelled using a binomial GLM to account for proportional dependent data, and McFadden's *R*^2^ index was used to assess predictive ability.

## 3. Results

We observed a total of 37 species encountering one another across 17 reefs in five regions of the Indo-Pacific (Christmas Island (Indian Ocean), Iriomote, Aceh, Bali, Philippines) before and after a mass coral bleaching event in 2016. Of the 37 species recorded, 32 were observed in ≥ five encounters across ≥ five focal individuals, which were our criteria for inclusion in statistical analyses, across 3770 encounters (electronic supplementary material, figure S1). The mean number of encounters within a species pair was 19.9 before and 15.5 after the coral mortality event for proximity data (max. = 130), 12.7 before and 9.1 after for signalling data (max. = 48), and 12.7 before and 8.4 after for chase data (max. = 40). Data were also reduced further to ensure species were present both in the heterospecific and conspecific encounters (*n* = 5–9; mean encounters per species pair = 9.43–34.72) to check that results were not driven by a greater range of species in one or other category (electronic supplementary material, figure S2). Due to their lower power, results from this matched dataset are only reported if they differ in effect direction and significance from the main dataset.

**Figure 1.**
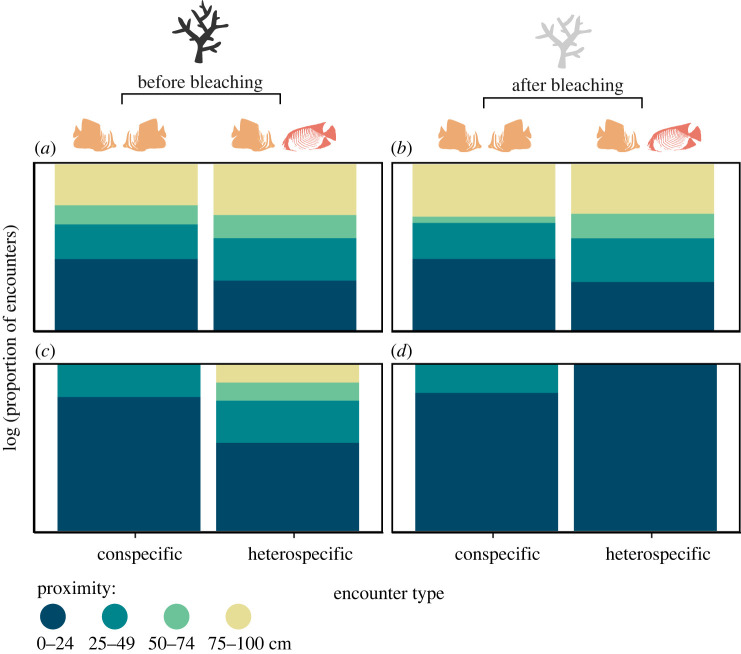
Approach proximity of individuals during an encounter before (*a,c*) and after (*b,d*) after mass coral mortality, for all encounter data (*a,b*) and for only those encounters that ended in overt agonism i.e. signalling or attack (*c,d*). Data include all species pairs with ≥ five samples across ≥ five individuals (*n* species before/after = 24/27, *n* encounters before/after = 2346/1334). (Online version in colour.)

*Prediction 1: Individuals approach heterospecifics less closely than conspecifics*. Individual median proximity during an encounter was lower for conspecifics than for heterospecifics, indicating a greater willingness to approach a conspecific before mass coral mortality (*Z* = 5.186, *p* < 0.001). This difference remained significant after mass coral mortality, although with a smaller effect size (*Z* = 3.771, *p* < 0.001; figure 1). Significant differences in approach proximity remained strong when the data were reduced to matched pairs only.

Proximity of encounters that involved signalling or escalated to aggression show a stark difference in the approach proximity of heterospecifics after coral mortality. While before the event, aggression was instigated at proximities up to 100 cm, after the event when resources were reduced, aggression was only initiated when heterospecifics were within 25 cm of each other. By contrast, conspecifics only engaged in aggression when within 50 cm of one another at both time points.

**Figure 2.**
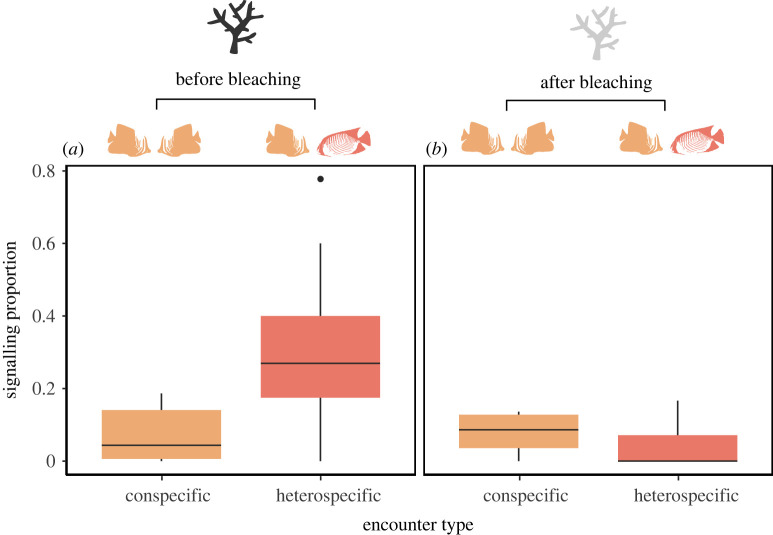
Proportion of aggressive encounters (less than 1 m) that were resolved through signalling, rather than chases (*a*) before and (*b*) after mass coral mortality. Data include all species pairs with ≥ five samples across ≥ five individuals (*n* species before/after = 15/6, *n* encounters before/after = 382/82). (Online version in colour.)

**Figure 3.**
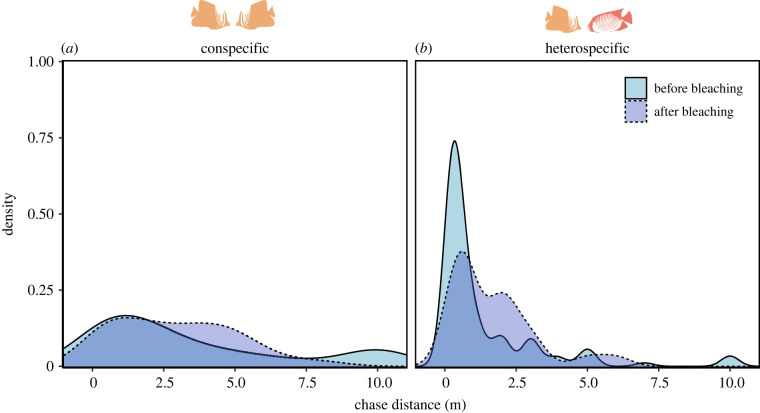
Chase distance distribution for conspecific and heterospecific encounters. Data include all species pairs with ≥ five samples across ≥ five individuals that were matched in both conspecific and heterospecific data (*n* species = 5/4, *n* encounters = 224/66). (Online version in colour.)

*Prediction 2: Signalling is more common between conspecifics than heterospecifics*. Our data do not support this prediction (figures 2 and 3). A significantly higher proportion of heterospecific than conspecific encounters involved signalling before mass coral mortality (*x*^2^ = 8.936 ± 0.092, 95% CI; *p* = 0.005 ± 0.001), which is the opposite pattern to the prediction. The mean proportion of encounters that escalated to chases across all species pairs was 0.72 for heterospecifics and 0.93 for conspecifics. After coral mortality, there was no significant difference between conspecifics and heterospecifics in signalling proportion (*x*^2^ = 0.361 ± 0.021; *p* = 0.730 ± 0.003) and mean proportions that escalated to a chase were 0.92 and 0.95, respectively. There were no significant differences in signalling frequency between heterospecifics and conspecifics for the smaller matched species group (electronic supplementary material, p3). Signalling proportion for conspecific encounters was not significantly different before and after bleaching (*x*^2^ = 0.195 ± 0.013; *p* = 0.859 ± 0.01), whereas heterospecific encounter signalling proportion changed significantly (*x*^2^ = 6.463 ± 0.018; *p* = 0.015 ± 0.001).

*Prediction 3: Attack intensity is more variable between heterospecifics than conspecifics*. Chase distances were more variable during heterospecific encounters than during conspecific encounters. Specifically, variation in chase distances across all encounters within a species pair was higher for heterospecifics than conspecifics before coral mortality, yet this difference was only significant when species were matched (table 1). However, after mass coral mortality, chase distance variation was very similar for both conspecific and heterospecific attacks (table 1).

**Table 1.** Comparison of variability in chase distances before and after mass coral mortality for a subset of species that have ≥ five samples (*n* species before/after = 12/6, *n* encounters before/after = 279/76), and species that are also matched in both conspecific and heterospecific encounters (*n* species = 5/4, *n* encounters = 224/66). Significant results are in italics.

	before ≥5	after ≥5	before matched	after matched
coefficient of variation				
conspecific	1.011	1.028	0.988	1.028
heterospecific	1.342	0.991	1.466	0.878
modified signed-likelihood ratio test for equality of CVs (MSLR)				
test statistic	2.523	0.031	*3.990*	0.279
*p-*value	0.112	0.860	*0.046*	0.598

According to MSLR, conspecifics did not change significantly in CV before and after coral mortality (MSLR 5 enc = 0.015, *p* = 0.903; MSLR matched = 0.086; *p* = 0.770). Heterospecific variation in chase distances did not change significantly but the effect size suggested a difference in the matched data (MSLR 5 enc = 1.388, *p* = 0.239; MSLR matched = 2.886; *p* = 0.089). Moreover, variation was no longer significantly different from conspecifics after mass coral mortality and heterospecific chases converged towards longer distances. Mean chase distance increased from 1.35 to 1.67 m (for conspecifics chase distance was stable at 3.46 m during both time points).

*Prediction 4: Proximity, signalling and variation in attack intensity can be predicted by phylogenetic relatedness or difference in body size.* For the proximity and signalling proportion models neither phylogenetic distance or body size difference predicted any of the behavioural data we tested significantly better than the intercept and effect sizes were low (Proximity: *β* phylogenetic distance = 0.35, *β* size difference = −0.01; adjusted *R*^2^ = 0.001; *p* = 0.338; Signalling proportion: *β* phylogenetic distance = 2.120, *β* size difference = 0.043; McFadden's *R*^2^ = 0.001). Chase variation had the strongest association with phylogeny and size difference, which explained 15% of the variation in the data (*β* phylogenetic distance = 0.12, *β* size difference = 0.11; adjusted *R*^2^ = 0.15; *p* = 0.141).

## 4. Discussion

Our results show that behavioural indicators of the competitor recognition process (i.e. cue–recognition–response) altered for heterospecific interactions, but not conspecific, following rapid environmental change. Specifically, after mass coral mortality, heterospecifics approached each other more closely before initiating aggression, fewer contests were resolved by signalling, and the distance chased—a proxy for energy invested—during attacks was more constant (and greater). We propose that these changes reflect a competitor recognition system that is mismatched with new environmental conditions, which could lead to suboptimal decision-making [41,42], exacerbating direct impacts of food scarcity. Our results therefore suggest that there is strong potential for disruption to competitor recognition processes to affect species coexistence under rapid environmental change.

Our findings suggest that established co-evolved cues and signals were less reliable indicators of defeat probability in an altered environment. The most plausible explanation for how and why the process has been compromised lies in the consequences of differential mortality rates across coral genera. Butterflyfishes shifted diet in response to coral mortality, which potentially led to reshuffling of established dominance hierarchies. The most aggressive species (e.g. *Chaetodon trifascialis, C. baronessa*) also had the most specialized diets [32], predominantly relying on corals of the *Acropora* genus; however, *Acropora* spp. were some of the most susceptible corals to bleaching-induced mortality at our field sites [7]. In response to this severe resource depletion, these fish species broadened their diet, leading to decreased dietary overlap [15]. At the same time, dietary breadth increased because of variation between individuals (niche variation hypothesis), reinforcing the idea that the lines of battle need to be redrawn because established niche partitioning was disrupted.

More fundamentally, while it seems that some heterospecifics recognize one another as competitors and subsequently engage in agonistic behaviour, recognition systems and the behaviours they elicit do not map on to phylogenetic relatedness. Although this finding is contrary to broad expectations [4,10], similar conclusions were drawn from a meta-analysis that found phylogeny was unable to predict heterospecific competitor recognition [14]. Differences in body size, which could be indicative of resource-holding potential [10], were also not predictive of the observed behaviours. Instead, these results offer support for the idea that recognition of heterospecifics is a function of resource overlap and is adaptive due to high levels of sympatry and niche overlap in butterflyfishes. Stable interspecific territoriality, which would require competitor recognition, has also been found in avian communities, adding weight to the idea that these dynamics are adaptive rather than simply mistakes in conspecific identification [13,17]. One caveat is that prior competition between the most closely related species could have led to divergent agonistic character displacement, which would ultimately replace aggression with avoidance [6] and be undetectable with our data.

### (a) Proximity to competitor

Individuals approached conspecifics more closely than heterospecifics, supporting the hypothesis that conspecifics are better able to estimate defeat probability [10], and only engaged in aggression at close proximity (less than or equal to 50 cm) regardless of resource availability. There is, however, an alternative and perhaps more parsimonious explanation for closer conspecific than heterospecific proximity—conspecifics are more likely to spatially aggregate at particular coral colonies because of high dietary overlap. If this hypothesis were supported, we would have expected to see even closer encounters between conspecifics as resources deplete, yet this was not observed. Counterintuitively, dietary overlap between butterflyfishes decreased after bleaching and there was greater dietary variation *within* species [15]. Therefore, in our system, if aggregation at a shared resource was the mechanism underlying proximity, conspecifics would be expected to reduce proximity after bleaching yet we observed no change in conspecific proximity before and after coral mortality.

Heterospecific encounters resulted in aggression across a wide range of proximities before coral mortality, yet after resources were depleted, only close encounters (less than or equal to 25 cm) initiated aggression. A mechanistic hypothesis for this change is that altered dietary overlaps [7,15] led to a mismatch between evolved neural templates to recognize competitors and the actual competitive threat that they posed. To counteract this mismatch, heterospecifics may have shifted, or added to, their competitor recognition cue to include direct feeding observations on a shared resource to confirm a competitor. Although we can only infer competitor recognition as the underlying driver of approach behaviour, we believe it is the most plausible explanation because alternative mechanisms, such as lowered risk of approach due to weaker competition, would generate the opposite pattern—heterospecifics would approach one another more closely than conspecifics, which was not observed. Size differences between species could also have offered an alternative explanation of greater clarity in competitive threat posed by heterospecifics; however, this variable was also not predictive of proximity, signalling frequency or attack variability.

### (b) Signalling to resolve contests

In contrast with the prediction of Peiman & Robinson [10], heterospecifics were more likely than conspecifics to resolve a contest with signals rather than escalate to attack—more than a quarter of heterospecific encounters ended with signalling. This result could reflect convergent agonistic character displacement [6] as there is likely to be strong selection pressure to recognize sympatric heterospecifics with high dietary overlap in a hyperdiverse system [10,43]. Models of ACD suggest that heterospecifics should undergo convergent character displacement, such as recognition of one another's signals, when exploitative competition is high relative to intraspecific competition [44], which is the case for coral reef fishes [45]. Indeed, evidence supports convergent ACD in sticklebacks as sympatric populations had higher aggression than allopatric populations [46,47]. We suggest that one way to explore this possibility further would be to target sister species combinations with recent secondary contact. Unfortunately, our sample sizes for sister species combinations were too small for this analysis because where they co-occurred, one sister species was always in low abundance.

After bleaching, signalling became less common between heterospecifics, with encounters escalating to chases in more than 90% of cases. The hypothesized mismatch between signals and the environment could reduce the truthfulness of signals to indicate defeat likelihood, leading to more attacks. However, it is worth bearing in mind that our method relied on observing signals that were visible to the human eye, and therefore we would miss any signals, and their changes, delivered by a different mode of communication (e.g. smell, sound and UV colours) [27].

### (c) Attack variability between individuals

Before coral mortality, there was greater variation in chase distance, and therefore in the energy expended, during heterospecific encounters than conspecific encounters. This result supports the hypothesis that individuals were less able to judge defeat probability against heterospecifics [10]. An alternative explanation could be that heterospecifics have larger differences in body size than conspecifics, offering an extra class for recognition, leading to less variability because contests can be resolved without an attack. However, body size difference was not predictive of chase variability, so this explanation is refuted.

When resources were reduced after coral mortality, the difference in attack variability dissipated, with individuals converging towards longer chase distances when faced with a heterospecific. Fish expended more energy in facing heterospecifics when resources were reduced, which follows predictions of cost-benefit economic defendability models because resources became more valuable due to their scarcity [10]. However, this change further suggests that the fish were less effective at identifying heterospecifics that were of minimal threat, investing energy in unnecessary attacks and adding weight to the explanation of compromised competitor recognition processes after resource loss.

### (d) Template mismatch as a mechanism for disrupted competitor recognition processes

We propose that changes in the behavioural indicators of the competitor recognition process for heterospecifics after coral mortality were driven by a mismatch of established neural templates with competitive threat following a redistribution of dietary overlaps and thus competition across species. Long-term effects of this mismatch depend on the extent to which templates can be updated throughout an individual's lifetime to reflect new ecological conditions, and even if templates are flexible, the time that it takes for templates to update. This potential is unresolved and is likely to vary across taxa from innate and inflexible, to flexible during sensitive imprinting periods, to flexible at any time [3]. For example, an experiment with damselfishes showed they were able to recognize new classes of competitors by observing the feeding behaviour of novel species [48]. By contrast, species recognition processes in fishes, albeit for reproduction, were compromised following increased turbidity and habitat degradation, presumably due to disrupted visual cues that could not be updated [27,49].

In our butterflyfish system, aggression per encounter decreased after the coral mortality event, which fits with optimal economic models, suggesting aggression had a higher relative cost [7]. Overall, we hypothesize that to counteract increased uncertainty over templates and to conserve limited energy by engaging in fewer attacks, individuals used a secondary cue of direct feeding on a disputed coral colony (*sensu* [48]), rather than relying solely on species ‘class’. This would explain why heterospecific individuals are closer before an attack. Following this logic, we would expect signalling to have reduced efficacy due to template mismatch and that when heterospecifics do attack, they consistently invest more in chase distance to ensure success, which fits with our observations. By contrast, we hypothesize that conspecific templates remained accurate as those individuals would maintain the biggest resource overlap despite dietary shifts, and potential crowding due to reduced resource availability would have been counteracted by increased niche differentiation between individuals [15]. Our results support this idea because there was no significant change in proximity, proportion of resolution by signalling, nor attack intensity variability during conspecific encounters.

An alternative route for disruption to the competitor recognition process, which could prove to be adaptive, is via a change in motivation. Evidence from ants suggests that once competitors are identified, motivation to attack can be influenced by environmental context, including group size and location [3]. Economic models suggest that cost-benefit analysis of aggressive territoriality is dependent on resource availability, with intermediate resource values associated with maximum aggression. High-resource abundance renders attacks unnecessary, while at very low resource availability, attacks carry an unacceptably high energetic cost [7,10]. Therefore, while the cues and templates could remain intact, the motivation to elicit a behavioural response could alter to reflect a new context [3].

## 5. Conclusion

Coral reef species are increasingly exposed to severe disturbance events [50] yet our understanding of how behavioural adjustments might mediate their response in a real-world context is in its infancy [7]. More broadly, as behavioural change is often the first response of an animal to environmental disturbance [41,51], the disruption of competitor recognition processes is concerning as it could have substantial knock-on effects at population, species and ecosystem levels [2,44], exacerbating the more direct detrimental effects of environmental change. Long-term, compromised abilities to recognize, and respond optimally to, competitors could even affect speciation processes by altering the selection pressure that underlies any ongoing agonistic character displacement [6,17]. Therefore, the role of competitor recognition systems in mediating interference competition and subsequent energetic costs could have substantial impacts on species coexistence within ecological communities under altered environmental conditions.

## Data Availability

Data are available from the Dryad Digital Repository: https://doi.org/10.5061/dryad.7d7wm3801 [52]. Supplementary material is available online [53].
